# Patient and health care system delays in the start of tuberculosis treatment in Norway

**DOI:** 10.1186/1471-2334-6-33

**Published:** 2006-02-24

**Authors:** Mohamed Guled Farah, Jens Henning Rygh, Tore W Steen, Randi Selmer, Einar Heldal, Gunnar Bjune

**Affiliations:** 1 Norwegian Institute of Public Health, Oslo, Norway; 2 Section for International Health, Institute of General Practice and Community Medicine, Faculty of Medicine, University of Oslo, Post box 1130 Blindern N-0318 Oslo, Norway; 3 Health and Welfare Agency, City of Oslo, Norway

## Abstract

**Background:**

Delay in start of tuberculosis (TB) treatment has an impact at both the individual level, by increasing the risk of morbidity and mortality, and at the community level, by increasing the risk of transmission. The aims of this study were to assess the delays in the start of treatment for TB patients in Oslo/Akershus region, Norway and to analyze risk factors for the delays.

**Methods:**

This study was based on information from the National TB Registry, clinical case notes from hospitals and referral case notes from primary health care providers. Delays were divided into patient, health care system and total delays. The association with sex, birthplace, site of the disease and age group was analyzed by multiple linear regression.

**Results:**

Among the 83 TB patients included in this study, 71 (86%) were born abroad. The median patient, health care system and total delays were 28, 33 and 63 days respectively, with a range of 1–434 days. In unadjusted analysis, patient delay and health care system delay did not vary significantly between men and women, according to birthplace or age group. Patients with extra-pulmonary TB had a significantly longer patient, health care system and total delay compared to patients with pulmonary TB. Median total delay was 81 and 56 days in the two groups of TB patients respectively. The health care system delay exceeded the patient delay for those born in Norway. The age group 60+ years had significantly shorter patient delay than the reference group aged 15–29 years when adjusted for multiple covariates. Also, in the multivariate analysis patients born in Norway had significantly longer health care system delay than patients born abroad.

**Conclusion:**

A high proportion of patients had total delays in start of TB treatment exceeding two months. This study emphasizes the need of awareness of TB in the general population and among health personnel. Extra-pulmonary TB should be considered as a differential diagnosis in unresolved cases, especially for immigrants from high TB prevalence countries.

## Background

The tuberculosis (TB) epidemiology in Norway has changed in the last 30 years. TB is now more likely to occur among immigrants (median age 31 years) than among those born in Norway (median age 72 years) [1,2]. The proportion of immigrants in the total population in 2003 was 7.3% [3]. DNA fingerprinting of bacterial strains indicated a low degree of recent transmission, implying that the majority of TB cases are due to reactivation of previous disease [4,5]. Compulsory screening for TB on entry has been implemented in Norway since the mid-1970s for persons from high TB prevalence countries who will stay more than three months [6].

Timely diagnosis of TB and start of treatment are important. Delay in start of TB treatment causes patients to have more advanced disease, more complications, higher mortality and more people being infected from each case [7-10]. It has been estimated that a patient with untreated smear-positive pulmonary TB may infect on average more than 10 patients annually and over 20 during the natural course of untreated disease until death [11]. To some extent, smear-negative pulmonary TB may also play a role in the spread of infection [12,13]. Delay in the start of TB treatment is relatively common both in high and low TB prevalence countries [14-19].

Delays are divided into patient delay, health care system delay and total delay. The health care system delay is assumed to be a larger problem than patient delay [20]. The problem of delay in the start of TB treatment has been described in several studies, which have indicated that the index of suspicion for diagnosing TB is low among health care providers [10,21-23]. There is not any universally accepted period for total delay from the onset of symptoms to the start of treatment for TB. Some authors have suggested that this period should not exceed one month [24], while others have suggested that a period of less than two months is acceptable [14]. Despite the importance of delay in the start of TB treatment, little is known about the extent of the delay in Norway. In one study, a median total delay of 15 weeks was found in 317 patients for whom information on duration of symptoms was available [25].

The aims of our study were to assess the delays in the start of treatment for TB patients in Oslo/Akershus region, Norway and to analyze risk factors for the delays.

## Methods

### Setting and study population

In Norway, there is compulsory nominative notification of all TB cases to the National TB Registry (NTR). Both suspected and confirmed cases have to be reported by clinicians. Laboratories of clinical microbiology are also required to report all isolates of *Mycobacterium tuberculosis *(*M. tb*). A total of 25 microbiological laboratories isolate *M. tb *from patient samples. The laboratory of the Norwegian Institute of Public Health functions as a national reference laboratory for TB. The pharmacy at the National Hospital is the only pharmacy distributing drugs for TB treatment, and it reports all TB prescriptions it handles to the NTR. The notification is therefore considered to be quite complete [26].

We identified the cases by reviewing notifications to the NTR. Notification forms and lab data were also reviewed. A total of 189 cases among persons aged 15 and above were reported to the NTR from July 1, 2003 to February 6, 2004. Of these, 83 cases were reported from the main Oslo and Akershus region hospitals and were included in this study.

We estimated the onset of the first TB symptoms from clinical case notes from hospitals and from referral case notes from primary health care providers. In addition, we have also reviewed the notification forms to the NTR.

For practical reasons, only information on delays in Norway was considered, but the patients (seven patients) who had delays abroad before arriving in Norway were included in the study. For this group, information on delays was considered from the arrival date in Norway.

### Definition

Patient delay was defined as the period from onset of symptoms related to TB to the date when the patient first contacted health services for those symptoms. Health care system delay was defined from the date of the patient's first contact with the health services for those symptoms to the date of the start of TB treatment. Total delay was defined as the sum of the patient delay and the health care system delay.

### Definition

Pulmonary TB case refers to disease affecting the lung parenchyma.  Extra-pulmonary TB case refers to TB outside the lung parenchyma. It is commonly found in the mediastinal lymph nodes, larynx, cervical lymph nodes, pleurae, meninges, central nervous system, spine, bones and joints, kidneys, pericardium, intestines, peritoneum and skin [27].

### Statistical analysis

We used the statistical package SPSS, version 11.0. To calculate the effect of variables such as sex, birthplace, site of the disease and age group on patient delay, health care system delay and total delay, we used multiple linear regression analysis. The data for the delays were skewed. Therefore, a logarithmic transformation was used to make the data more normally distributed. P values of less than 0.05 were considered statistically significant.

This study received approval from the Regional Ethics Committee for Medical Research, The Norwegian Data Inspectorate and the Norwegian Directorate for Health and Social Affairs.

## Results

A total of 83 patients were included in this study. Of these, 71 were foreign-born (86%). The median and mean periods of residence in Norway for the foreign-born patients were 2.5 and 5.1 years respectively. The median age for all patients was 32 years. Among those born in Norway, the median age was 57 years while among the foreign-born, the median age was 30 years.

There were 57 pulmonary cases and 26 extra-pulmonary cases. Of the 83 cases included in the study, 78 were new cases and five were previously treated cases. Of the 57 pulmonary cases, 52 were culture-positive, three were culture negative and results were not known for two. Information on smear status for pulmonary cases was available for 36 cases. Of these, 19 were smear-positive and 17 were smear-negative.

Susceptibility testing for the main TB drugs was done for most of the culture-positive patients (88%). Two patients had multi-drug resistant TB (MDR-TB), i.e. resistance to both isoniazid (INH) and rifampicin. Both were foreign-born patients. An additional two patients had isolated INH resistant strains.

The most frequent symptoms reported by the patients were cough, fever and weight loss (Table 1). Other symptoms were night sweat and general malaise. For patients with extra-pulmonary TB, the most frequent symptoms were gland enlargement, headache and pain in the neck and back.

**Table 1:** Symptoms* reported by patients with pulmonary tuberculosis (N = 57)

Symptoms	Number of patients (%)
Cough	42 (74%)
Productive	17 (30%)
Dry	25 (44%)
Weight loss	25 (44%)
Fever	20 (35%)
Night sweat	18 (32%)
Hemoptysis	9 (16%)
Dyspnea	11 (19%)
Chest pain	14 (25%)
General malaise	15 (26%)
Other	28 (49%)
No symptoms reported	5 (9%)

* For most patients, two or more symptoms were reported

For the patient, health care system and total delays there were missing data for 21, 12 and 16 cases respectively. Of the 83 cases included in this study, 61 cases had all the information on patient, health care system and total delays.

The median patient, health care system and total delays for all patients were 28, 33 and 63 days respectively (Table 2). Of the pulmonary cases, the median patient, health care system and total delays were 21, 22 and 56 days respectively. For the extra-pulmonary cases, the median patient, health care system and total delays were 42, 42 and 81 days respectively. The median health care system delay was longer than patient delay in patients born in Norway.

**Table 2:** Patients characteristics for patient, health care system and total delays

	N	Patient delay	Health care system delay	Total delay
				
		Median delay/ (range) in days*		
Sex				
Women	39	28/ (2–217)	35/ (2–182)	77/ (9–343)
Men	44	25/ (2–154)	22/ (1–378)	60/ (4–434)
Birthplace				
Norway	12	21/ (3–84)	49/ (21–160)	56/ (35–147)
Abroad	71	28/ (2–217)	28/ (1–378)	67/ (4–434)
Site of the disease				
Pulmonary	57	21/ (2–158)	22/ (1–378)	56/ (4–434)
Extra- pulmonary	26	42/ (10–217)	42/ (4–224)	81/ (14–378)
Age group				
15–29 yrs	35	28/ (2–217)	35/ (1–224)	70/ (4–378)
30–44 yrs	29	28/ (3–158)	21/ (2–119)	49/ (16–259)
45–59 yrs	11	49/ (5–126)	53/ (4–378)	112/ (9–434)
60+ yrs	8	18/ (3–28)	49/ (25–70)	65/ (42–214)
All	83	28/ (2–217)	33/ (1–378)	63/ (4–434)

*Including missing data

For those with information on smear status, the median delays for patient, health care system and total for smear-positive pulmonary cases were 28, 14 and 45 days respectively. For the smear-negative pulmonary cases, the median patient, health care system and total delays were 14, 46 and 60 days.

The site of the disease was the only significant predictor of both patient, health care system and total delay in unadjusted and adjusted analysis, with delays in patients with extra-pulmonary TB varying from two to three times the delays in patients with pulmonary disease (Table 3). The age group 60+ years had significantly shorter patient delay than the reference group aged 15–29 years when adjusted for multiple covariates. Also, in the multivariate analysis patients born abroad had significantly shorter health care system delays than patients born in Norway.

**Table 3:** Relative treatment delays estimated by the exponential of regression coefficients from multiple linear regression. Dependent variables: log (patient delay), log (health care system delay) and log (total delay).  (95 % confidence intervals)

	Patient delay		Health care system delay		Total delay	
			
	Univariate	Multivariate†	Univariate	Multivariate	Univariate	Multivariate
						
	Relative delay	Relative delay	Relative delay	Relative delay	Relative delay	Relative delay
Sex						
Women‡	1.00	1.00	1.00	1.00	1.00	1.00
Men	0.68 (0.37–1.27)	0.71 (0.39–1.29)	0.76 (0.44–1.34)	0.84 (0.49–1.45)	0.77 (0.48–1.24)	0.81 (0.50–1.29)
Birthplace						
Norway‡	1.00	1.00	1.00	1.00	1.00	1.00
Abroad	1.26 (0.44–3.62)	0.56 (0.17–1.89)	0.46 (0.19–1.12)	0.32 (0.11–0.94)	0.94 (0.47–1.88)	0.81 (0.37–1.78)
Site of the disease						
Pulmonary‡	1.00	1.00	1.00	1.00	1.00	1.00
Extra-pulmonary	2.44 (1.31–4.54)	2.97 (1.57–5.61)	1.92 (1.08–3.42)	2.04 (1.13–3.68)	1.80 (1.11-2.91)	1.81 (1.10–2.98)
Age group						
15–29 yrs‡	1.00	1.00	1.00	1.00	1.00	1.00
30–44 yrs	0.99 (0.49–2.02)	0.94 (0.48–1.82)	0.60 (0.33–1.12)	0.61 (0.34–1.10)	0.79 (0.47–1.35)	0.80 (0.47–1.35)
45–59 yrs	1.69 (0.66–4.38)	1.90 (0.79–4.58)	1.54 (0.61–3.85)	1.70 (0.70–4.13)	1.52 (0.72–3.20)	1.59 (0.77–3.28)
60+ yrs	0.55 (0.15–2.10)	0.21 (0.05–0.99)	1.40 (0.46–4.27)	0.41 (0.10–1.63)	1.13 (0.47–2.70)	0.87 (0.32–2.35)

†In the multivariate analysis, all variables in the univariate analysis were included.

‡ Reference category.

Of the 83 patients included in the study, 66 patients were hospitalized before start of treatment. The median and mean period between hospital admission and treatment start for those hospitalized before start of the TB treatment was 4 (1–82) and 18 days, respectively.

Of the 37 TB cases with more than two months of total delay, 20 were pulmonary cases and 17 were extra-pulmonary cases. Of these 33 (89%) were born abroad, whereas 86 % of all cases were foreign-born.

### We have studied in detail 15 of the 37 patients with a total delay of more than two months, for whom more specific information was available

1. Nine patients had received several courses of antibiotics from primary health care providers before the start of TB treatment. Some had family members with a history of previous TB. They were sent for further examination at the hospital only after the courses of antibiotics did not help.

2. Four patients had long total delay mainly due to difficulties in isolating bacilli, difficulties in making TB diagnosis and negative results of the initial tests done (sputum and x-ray were negative). For some, the x-ray did not give a clear indication for TB.

3. Two patients had concomitant diseases such as asthma and Morbus Crohn that caused delays in suspecting TB disease.

All 15 patients, except one, were born abroad. Thirteen came from countries with a high prevalence of TB. The majority of all TB patients in this study were born abroad. Thus, even though those patients born abroad had shorter health care system delays, most patients with long total delays were also born abroad.

### We have also studied in detail 4 of the 14 patients with less than one month total delay, for whom more specific information was available

1. There was suspicion of TB when the patient first visited the primary health care provider. Still, the patient was first treated with antibiotics for presumed pneumonia.

2. The delay was short because the patient had been treated for TB before and also had symptoms related to TB.

3. The patient was admitted to the hospital for other reason than TB disease. There was no history of TB in the family, but an x-ray suggested TB.

4. At hospital admission, there was a strong suspicion of TB due to the patient's country of origin (a high TB prevalence country) and previous TB in the family.

## Discussion

Our study has indicated a median total delay of 63 days from the onset of symptoms to the start of TB treatment for all 83 patients. In our study, the median health care system delay for all patients included in the study exceeded the median patient delay. But among those born in Norway, health care system delay was more than twice as long as patient delay. Many studies have found that patient delays were longer than health care system delays [28,29]. Other studies have found the opposite [18,30,31]. The main reason for the delay in the studies in which the health care system delay was longer than the patient delay was failure of accurate diagnosis at the initial presentation. In our study, the main reason for the long total delay was that the health care providers did not initiate specific TB examination despite symptoms such as cough, weight loss and night sweat. TB examination was initiated only after unsuccessful antibiotics treatment. Some patients had other diseases such as asthma that made it more difficult to suspect TB.

The health care system delay for those born in Norway was twice as long as for those born abroad. One reason for the longer health care system delay for those born in Norway could be that the index of suspicion for TB might be lower for this group, since TB incidence among those born in Norway is low. Some studies from other low TB prevalence countries have shown that there is a greater awareness among health care providers of the risk of TB for those born abroad [32,33].

Most TB patients born in Norway are in older age. Many of them have co-existing illness. This makes TB diagnosis difficult and thus causes delay [34,35]. Older patients may present with respiratory symptoms less often than younger patients [21,28,34-37]. This also explains why the condition of many of the older patients could deteriorate without TB being considered. Awareness for TB is needed for this group.

Some reasons that might explain the longer patient delays for those born abroad are stigma, fear of deportation, lack of TB knowledge, language problems and cultural differences in the interpretation of signs and symptoms [38]. Some authors have suggested that social and cultural factors may influence patients' decision to seek help. In many cultures, the patient's problem is compounded by the social stigma of TB, a fear that may contribute to a long delay in seeking professional care and even to abandonment of treatment [39]. In our study, 86% of the patients were foreign-born and the majority of them came from countries with high TB prevalence, in which stigma may play a major role.

In the multiple regression analysis for patient, health care and total delay, extra-pulmonary site was the only factor that was significant in both univariate and multivariate analysis. It was not surprising that patients with pulmonary TB had shorter delay than patients with extra-pulmonary TB. Patients with extra-pulmonary TB may have a variety of symptoms and are difficult to diagnose. In our study, 17 of the 37 TB cases with more than two months of total delay were extra-pulmonary TB cases. From a public health perspective, these cases have less consequence for the spread of the disease, as cases of extra-pulmonary TB are rarely infectious. However, they may cause a diagnostic challenge for health personnel and could easily be overlooked. For the patients, delay in the start of TB treatment can result in increased severity and mortality [40,41]. But as shown in our study, there were several cases with pulmonary TB, some with symptoms such as cough, weight loss and night sweat that had total delays of more than 90 days.

In our study, we have investigated the median time between hospital admission and the start of TB treatment. Although the data were not complete, we found that around 80% of the patients included in this study were hospitalized before the start of TB treatment. The median time between hospitalization and the start of TB treatment was four days (1–82). Studies from Washington, DC, United States of America (USA) and St. Louis, USA have also found a median time of six days between the hospitalization and the start of TB treatment [34,42]. In these studies, delays in the start of TB treatment were longer than delays in the initial suspicion of TB. It was also the case even for patients with smear-positive disease. This was explained by the fact that health care providers waited for confirmation by culture results. Delaying the start of TB treatment could increase transmission and mortality of TB, but early initiation of treatment without culture verification might carry the risk of starting treatment on the basis of a wrong diagnosis [43,44]. In our study, only three patients had no information about culture status. According to the new Norwegian manual for TB control and prevention [6], it is recommended that treatment could either be started on clinical suspicion of TB or one can wait for culture results if sputum smear result is negative. In our study, some health care providers may have taken the latter approach. It explains also that smear-negative pulmonary cases had longer health care system delay compared to smear-positive cases. In our study, the median health care system delay for smear-positive pulmonary cases was 14 days.

An advantage of our study was that we have analyzed delays for both pulmonary TB and extra-pulmonary TB cases. Most studies that have studied delays have evaluated delays for pulmonary TB only. The two sites of the disease are different with regard to symptoms and diagnosis. However, both reflect the circulation of the *M. tb *in the population.

One limitation of our study was that primary health care and hospital notes were often incomplete regarding delays, especially for the patient delay. For some patients, the referral letter was missing. This made it difficult to find the exact date when the patient first contacted a health care provider for symptoms related to TB. Since we have collected the date of the onset of symptoms from different sources, there was a potential for discrepancies in the estimated dates for individual patients. We have made efforts to minimize these potential discrepancies by validating the data through different sources for each patient. Data on delays were missing for some cases. This could influence the results of our study. But when we evaluated separately all cases with complete information on delays (61 cases) and all cases included in the study with missing data on same types of delay (83 cases), the univariate and multivariate analysis for both gave similar results. So the missing data on same types of delay did not affect the overall results of this study. Another limitation was that we used only the delays in Norway for some foreign-born (seven patients) for practical reasons. They were included in the study, but the data for delays abroad were not included. Although this affected very few cases, it is possible that we have underestimated the actual delays for this group.

## Conclusion

A high proportion of patients had total delays in start of TB treatment exceeding two months. This study emphasizes the need of awareness of TB in the general population and among health personnel. Extra-pulmonary TB should be considered as a differential diagnosis in unresolved cases, especially for immigrants from high TB prevalence countries.

## List of abbreviations

CI Confidence interval

INH Isoniazid

Log Logarithm

MDR-TB Multi-drug resistant TB

NTR National Tuberculosis Registry

M. tb Mycobacterium tuberculosis

TB Tuberculosis

USA United States of America

## Competing interests

The author(s) declare that they have no competing interests.

## Authors' contributions

MGF is the primary author responsible for designing of the study, collection of the data, analysis and interpretation of the results and writing of the draft and final manuscript and is corresponding author. JHR, TWS, RS, EH and GB have participated the interpretation of results and writing of the draft and final manuscript. All authors read and approved the final manuscript.

## Pre-publication history

The pre-publication history for this paper can be accessed here:


